# Attenuating Fibrotic Markers of Patient-Derived Dermal Fibroblasts by Thiolated Lignin Composites

**DOI:** 10.1021/acsbiomaterials.1c00427

**Published:** 2021-05-03

**Authors:** Jorge A. Belgodere, Dongwan Son, Bokyoung Jeon, Jongwon Choe, Anna C. Guidry, Adam X. Bao, Syed A. Zamin, Umang M. Parikh, Swathi Balaji, Myungwoong Kim, Jangwook P. Jung

**Affiliations:** ‡ Department of Biological Engineering, Louisiana State University, Baton Rouge, Louisiana 70803, United States; § Department of Chemistry and Chemical Engineering, Inha University, Incheon 22212, Republic of Korea; ⊥ Department of Pediatric Surgery, Texas Children’s Hospital and Baylor College of Medicine, Houston, Texas 77030, United States

**Keywords:** antioxidation, fish gelatin, lignosulfonate, fibrosis, wound healing

## Abstract

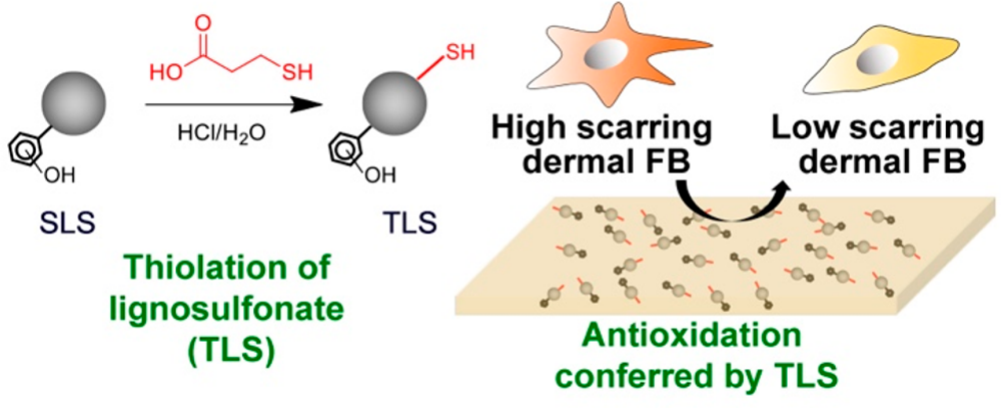

We report the use of phenolic functional groups of lignosulfonate to impart antioxidant properties and the cell binding domains of gelatin to enhance cell adhesion for poly(ethylene glycol) (PEG)-based scaffolds. Chemoselective thiol–ene chemistry was utilized to form composites with thiolated lignosulfonate (TLS) and methacrylated fish gelatin (fGelMA). Antioxidant properties of TLS were not altered after thiolation and the levels of antioxidation were comparable to those of *L*-ascorbic acid. PEG-fGelMA-TLS composites significantly reduced the difference in *COL1A1*, *ACTA2*, *TGFB1*, and *HIF1A* genes between high-scarring and low-scarring hdFBs, providing the potential utility of TLS to attenuate fibrotic responses.

Reactive oxygen species (ROS) are generated in aerobic cells either as byproducts during mitochondrial electron transport or by oxidation of metabolites.^1^ Although ROS are considered toxic agents to disrupt cell division and induce apoptosis, ROS serves as signaling molecules when tightly regulated and then are taken up by the cell.^2−4^ Thus, the critical balance of intracellular ROS is of vital importance for cell survival with the increase in extracellular ROS most likely leading to cellular apoptosis, unwanted intracellular signaling, and/or genotypic changes that can manifest as phenotypic changes.

Because of the naturally occurring polyphenolic structures in lignin, the OH functional groups possess the ability to neutralize radicals, including those present at a wound site.^5,6^ Recently, the incorporation of lignosulfonate into the collagen matrix was demonstrated to yield enhanced mechanical properties while avoiding cytotoxic and immunogenic responses for use as *in vitro* scaffolds or *in vivo* tissue repairs.^7^ To reduce the variation of biochemical and mechanical properties of lignin-based scaffolds, utilization of highly efficient, covalent cross-linking chemistry was required to form composites that support tissue engineering applications.

Here, we employed poly(ethylene glycol) (PEG)/methacrylated fish gelatin (fGelMA) composites to assess the antioxidant capacity of lignosulfonate. Although lignosulfonate and fish gelatin have not been widely used in tissue engineering applications, their applications have been increasingly reported as a sustainable and economic source of engineered biomaterials.^8−16^ A key difference between fish and mammalian gelatins is a lower proline and hydroxyproline content resulting in a decrease in mechanical properties, altering the sol–gel transition temperature allowing fish gelatin to remain liquid at room temperature.^17−19^ Methacrylation and thiol–ene chemoselective reaction offer a controllable cross-linking methodology to confer enhanced stiffness, whereas gelatin still provides integrin binding sites for tissue scaffolds.^20^ Thus, employing both lignosulfonate and fish gelatin provides a sustainable feedstock while valorizing currently underutilized biological materials.

Of several routes of integrating the biomaterials into a scaffold, we functionalize lignosulfonate with 3-mercaptopropionic acid (MPA) to form thiolated lignosulfonate (TLS) allowing conjugation of the lignosulfonate to diacrylated PEG (PEGDA) and fGelMA via thiol–ene chemistry,^21,22^ which is one of the most widely utilized methods for high yields, experimentally straightforward methods, and having no byproducts.^23−25^ The reaction forms thioether bonds that must use either base-catalyzed electron-deficient alkenes or by a radically initiated reaction with UV irradiation or thermolysis.^26,27^ Because neither PEGDA nor TLS provide cell adhesion sites, we formed composites with fGelMA (Figure S1) for cultures of patient-derived dermal fibroblasts. We characterized the functionalization, antioxidant capacity and mechanical properties of PEG-fGelMA-TLS composites and the modulation of fibrotic gene (*COL1A1*, *ACTA2*, *TGFB1*, and *HIF1A*) expression.

^1^H NMR (nuclear magnetic resonance) and FT-IR (Fourier-transform infrared) spectroscopies of SLS and TLS (Figures S2 and S3) were used to confirm the thiolation of SLS (Figure 1a, note that the esterification sites and their number were exemplified due to the structural complexity of lignosulfonate). The appearance of a broad peak corresponding to the protons of the −S–CH_2_– group were centered between δ 1.9–3.2 ppm. Furthermore, the intensity of the broad peak for aliphatic–OH (δ 2.8–4.3 ppm) was reduced when comparing SLS to TLS. The emergence of the intense band at near 1700 cm^–1^ in FT-IR spectra (Figure S3) assigned to stretch mode of the C=O in ester confirm the successful esterification reaction between SLS and MPA.^28^ To further quantitatively confirm the extent of thiolation with a higher degree of specificity, we performed ^31^P NMR spectroscopy,^29^ resulting in peaks of phenolic–OH and aliphatic–OH as evidenced in Figure 1b. The chemical shifts at 132.2 ppm was assigned to the product of TMDP (2-chloro-4,4,5,5-tetramethyl-1,3,2-dioxaphospholane) hydrolysis and the lignin products^29^ since the lignin products do not contain any phosphorus atom. The NMR spectra show peaks at 145.6–149.8 ppm (assigned to aliphatic–OH) and at 137.6–144.4 ppm (assigned to phenolic–OH). Quantitative analysis using the internal standard (OH group of cyclohexanol) peak at 145.0 ppm with known concentration allowed us to determine the degree of thiolation, where the changes of the integrated peak intensity of phenolic–OH and aliphatic–OH before and after thiolation were 0.62 and 0.35, respectively. These results strongly suggest that aliphatic–OH groups were more actively involved in the incorporation of thiol compared to phenolic–OH groups, which would be advantageous to maintain antioxidant capacity of lignosulfonate. Using Ellman’s assay, the incorporated thiol groups were quantified, confirming that the thiol concentration was precisely controlled by tuning reaction stoichiometry as evidenced by the thiol concentration of three different batches of TLS at different stoichiometric ratios of SLS to MPA (Figure 1c). When compared to the SLS control, concentrations of thiols were increased almost 10-fold without compromising batch to batch replicability. In sum, TLS functionalization is tunable and primarily occurs at aliphatic OH groups.

**Figure 1:**
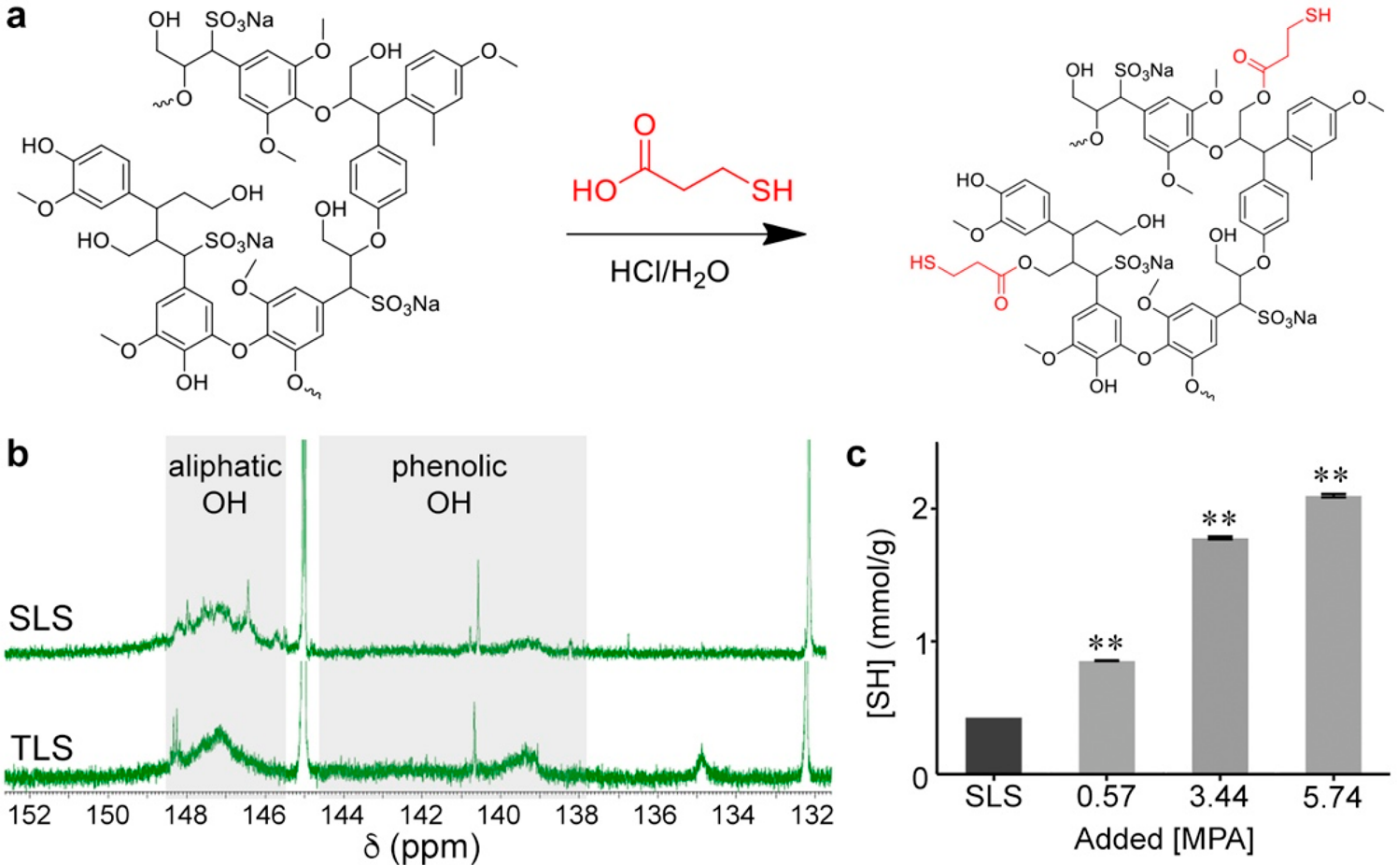
Schematic and assessment of SLS thiolation. (a) Thiolation of SLS to form TLS. (b) ^31^P NMR (in CDCl_3_) spectra of SLS or TLS via TMDP (2-chloro-4,4,5,5-tetramethyl-1,3,2-dioxaphospholane) hydrolysis. (c) Thiol concentrations of TLS samples prepared with a varied stoichiometry determined by Ellman’s assay. [MPA] is defined as the number of moles (mmol) of added MPA per 0.1 g of SLS; **p < 0.01, ANOVA Dunnett’s *post hoc* test, mean ± standard deviation (SD), *n* = 3.

To evaluate the antioxidant potential for SLS and TLS, we utilized two common assays to assess antioxidation using DPPH (2,2-diphenyl-1-picrylhydrazyl) and the reduction of Cu^2+^ to Cu^+^ (TAC, total antioxidant capacity). We tested the extent of DPPH inhibition by SLS and TLS at multiple concentrations, from 1 to 5 mg/mL. As shown in Figure 2a, TLS showed significantly higher DPPH inhibition in comparison to SLS and significantly lower DPPH inhibition in comparison to *L*-ascorbic acid (*L*-asc) at all three concentrations. In Figure 2b, similar trends were observed, but the difference between SLS and TLS decreased. SLS also exhibited variability in the capacity of DPPH inhibition, which may be due to incomplete dissolution in the mixture of ethanol and water up to 30 min at higher concentrations (Figure 2a). However, the variability disappeared after 24 h of incubation, indicating that SLS is fully dissolved and radical scavenging groups are more available. In Figure 2c, TLS exhibited higher TAC (reducing Cu^2+^ to Cu^+^) than that of SLS at 1 and 2 mg/mL. At 2 and 5 mg/mL, TLS showed comparable TAC in comparison to *L*-asc. Apparently, TLS is a better scavenger than SLS because thiols can also scavenge radicals. As evidenced in Figure 1b, thiolation occurred mainly on aliphatic OH. These results support that TLS possesses a dual mode of radical scavenging with phenolic groups of lignosulfonate and thiolated aliphatic chains of lignosulfonate.

**Figure 2:**
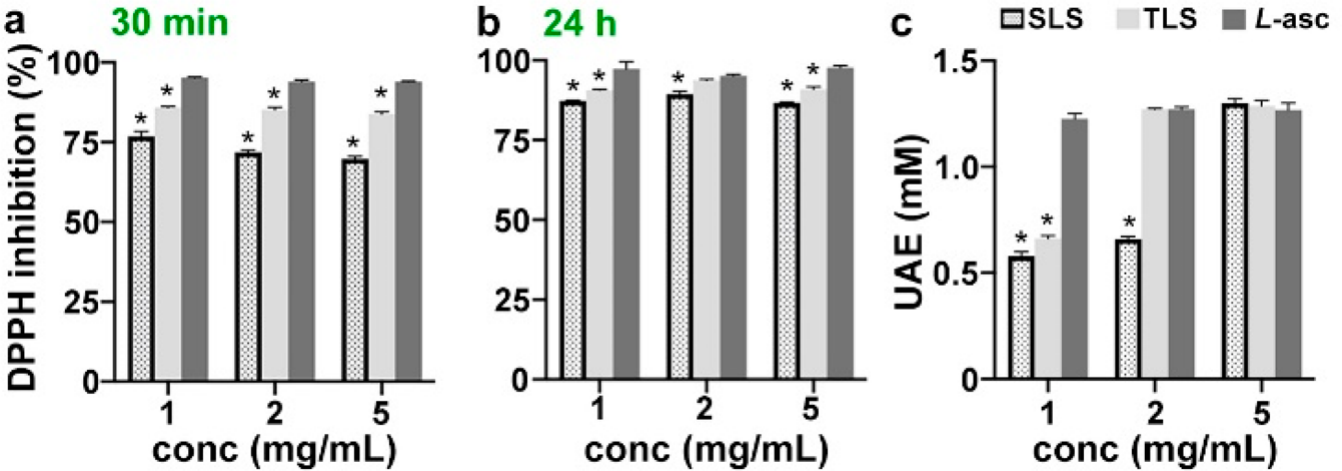
Independent antioxidant assays to assess radical scavenging capacity of TLS. DPPH assay of SLS, TLS and *L*-ascorbic acid for (a) 30 min and (b) 24 h. (c) TAC assays with the same samples. *p < 0.01, ANOVA Dunnett’s *post hoc* test, mean ± SD, *n* = 3.

Methacrylation of fGelMA was assessed using ^1^H NMR spectroscopy with D_2_O as a solvent (Figure S4).^30,31^ Pristine fGel shows a peak at 3.0 ppm, which is assigned to the proton of the primary amine in lysine. Upon the reaction with GMA (glycidyl methacrylate), the intensity of the peak significantly decreased, indicating the disappearance of primary amines by the reaction. Simultaneously, the emergence of two peaks at 6.1–6.2 ppm and 5.7–5.8 ppm was observed, assigned to protons of alkene in the methacrylate. The degree of the lysine functionalization was calculated with integrated intensities of the peak of primary amine in lysine and the peak of phenylalanine at 7.2–7.5 ppm. The peak intensity of the phenylalanine was used as a reference to estimate the degree of functionalization by comparing the peak intensities of lysine amine before and after the reaction. In multiple performed reaction batches, the degree of functionalization was estimated at least 0.77. Using oscillating rheometry, we measured viscosity of fGelMA at 40 and 200 mg/mL. As shown in Figure 3a, viscosities of fGel were 0.6 Pa s (40 mg/mL) and 3.2 Pa s (200 mg/mL), whereas those of fGelMA were 0.5 Pa s (40 mg/mL) and 1.14 Pa s (200 mg/mL) at room temperature. Because of such low viscosity at room temperature, we were able to form composites including gelatin without adding acetic acid^32^ or heating^33^ in the cases of applying porcine gelatin. As shown in Figure S5, the viscosity of porcine GelMA (pGelMA, 180 mg/mL) was several orders of magnitude higher than that of fGelMA (200 mg/mL). The reduced viscosity of fGelMA compared to fGel observed at high concentration of 200 mg/mL can be attributed to the removal of impurities in fGel by dialysis after methacrylation or by the reduction of the amount primary amine group, which induces interchain interaction with carboxylic acid along the chains, instead of changes in chemical nature of gelatin chains.

**Figure 3:**
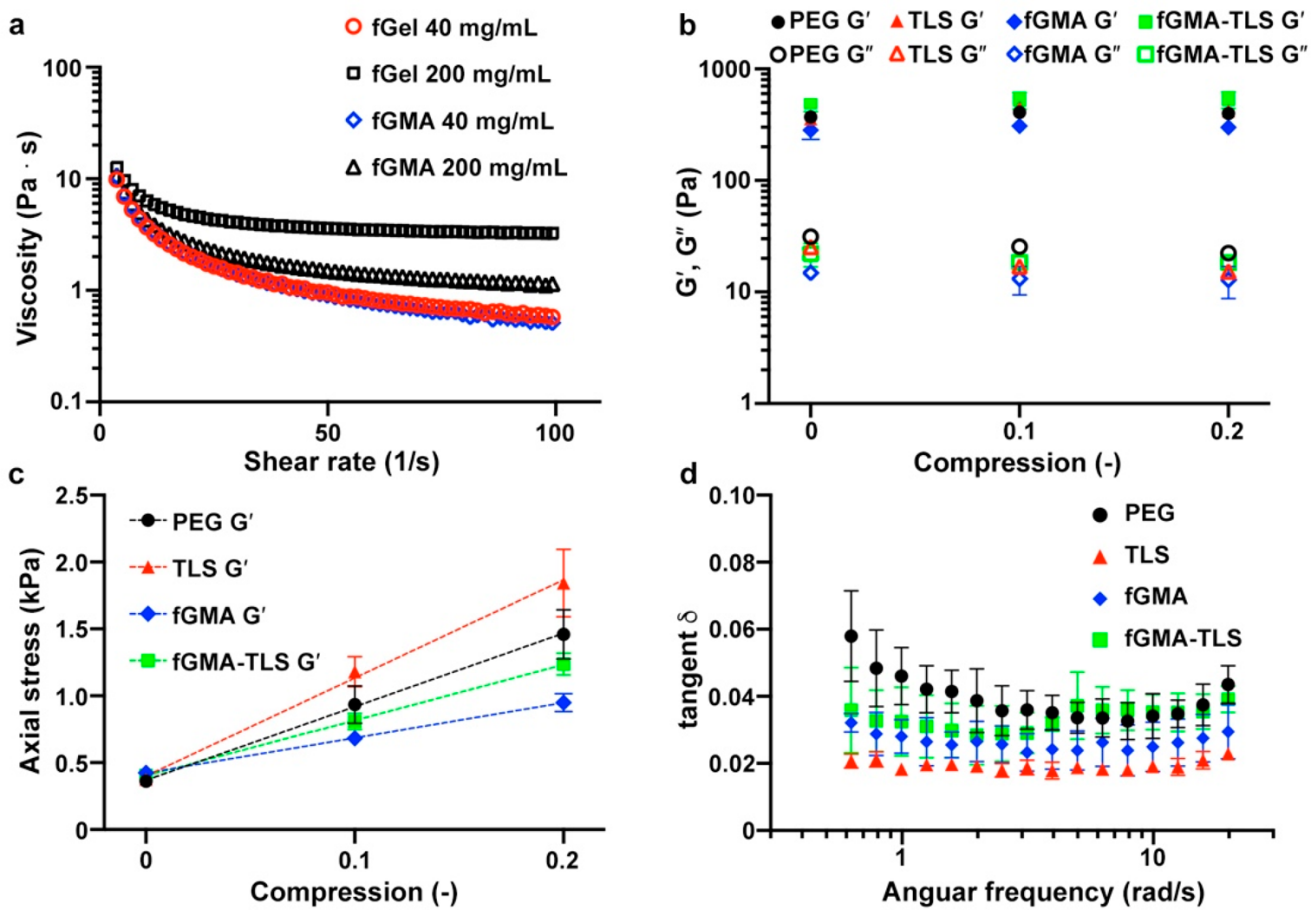
Assessment of rheological properties. (a) Viscosity of fGel (before methacrylation) and fGMA (fGelMA, after methacrylation) at 40 and 200 mg/mL. (b) Storage (*G*′) and loss (*G*″) moduli of PEG (40 mg/mL), TLS (PEG 40 mg/mL and TLS 5 mg/mL), fGMA (PEG 40 mg/mL and fGelMA 40 mg/mL, 4:1 (v/v)) and fGMA-TLS (PEG 40 mg/mL and fGelMA 40 mg/mL (4:1 (v/v)) with TLS 5 mg/mL) at compression varying from 0 to 20%. All composites were cross-linked with 5 mg/mL LAP (lithium phenyl-2,4,6-trimethylbenzoylphosphinate). (c) Axial stresses were plotted against compression varying from 0 to 20%. Details of trend lines are summarized in Table 1. (d) Loss tangent (δ) of the PEG composites from 0.62 to 19.9 rad/s. Mean ± SD, *n* = 3.

Composite hydrogels consisting of PEGDA, TLS, and/or fGelMA were formed with photo-cross-linking as depicted in Figure S1. The PEG-TLS composites were prepared at a 1:0.5 alkene:thiol ratio, with a PEGDA concentration set at 40 mg/mL. We attempted higher alkene:thiol ratios, 1:1 for example, however, had issues with cross-linking due to the ability of lignin to absorb UV light, resulting in effective cross-linking of only the surface region. With the maximal antioxidant capacity from TLS and the interference by higher concentration of TLS (>5 mg/mL) during photo-cross-linking of PEG composites,^34^ we applied TLS at 5 mg/mL throughout the rest of our experiments.

To further delineate changes of mechanical properties upon incorporating TLS and/or fGelMA, we measured *G*′ (storage modulus, elasticity) and *G*″ (loss modulus, viscosity) under various conditions. Complete contact and certain axial force are required to measure viscoelasticity using a rheometer. However, we often observed that altering axial force can result in different *G*′ and *G*″, thus we sought to extract a stress–strain curve from axial stress-compression data.^35^ As shown in Figure 3b, all *G*′ were overlapped from around 0.3 to 0.5 kPa. Additionally, *G*′ and *G*″, as a function of compression, did not show significant difference at different compression varying from 0 to 0.2 (or 20%). When these data were put in axial stress vs compression (Figure 3c), the slope yielded a modulus of elasticity and those values are summarized in Table 1. The incorporation of TLS increased the elasticity from 5.5 to 7.3 kPa in the absence of fGelMA, whereas the incorporation of fGelMA decreased without TLS decreased the elasticity from 5.5 to 2.6 kPa. Once we added 5 mg/mL TLS and 40 mg/mL fGelMA to 40 mg/mL PEG, the stiffness of the composite was increased to 4.2 kPa. This estimation is predictable, as evidenced by high *R*^2^ values, and the modulation of the stiffness of PEG composites is feasible by modulating the quantity of TLS and/or fGelMA incorporated. As shown in Figure 3d, loss tangent of all the PEG composites varied in such a narrow range from 0.06, equivalent to 3.43° and indicative of covalently cross-linked, highly elastic hydrogels when subject to angular frequency from 0.628 to 19.9 rad/s at 2% strain. PEG exhibited the highest loss tangent, whereas the incorporation of TLS conferred the lowest loss tangent and minimal changes throughout the range of frequencies we tested (PEG-TLS). Incorporating fGelMA or fGelMA-TLS showed similar loss tangent and levels between those of PEG and TLS.

**Table 1:** Elastic Modulus Estimated by the Slope of Axial Stress vs Compression

	slope (elastic modulus, kPa)	intercept (kPa)	*R*^2^
PEG only	5.5	0.2	0.9422
TLS (PEG-TLS)	7.3	0.4	0.9512
fGMA (PEG-fGelMA)	2.6	0.4	0.9585
fGMA-TLS (PEG-fGelMA-TLS)	4.2	0.4	0.9760

fGelMA was added to the PEG-TLS precursor solutions and cast prior to UV cross-linking to synergistically enhance cell attachment of hdFBs (human dermal fibroblasts). Stock solutions were prepared for PEG and fGelMA at 50 and 200 mg/mL, respectively, and mixed at different volume ratios. The amount added was varied (Figure 4a–d) to introduce the minimal amount of fGelMA not to interrupt the antioxidation by TLS, whereas maintaining the biophysical properties by PEG. As expected, PEG-only composites showed minimal cell attachment (Figure 4a). Increasing fGelMA further resulted in enhanced cell attachment with an 80:20 mixture exhibiting a hdFB monolayer across the surface of the composite (Figure 4c). Although a 50:50 (Figure 4d) provided more binding sites for cell attachment, the distribution of hdFBs was less homogeneous than that of the composite with 80:20 mixture.

**Figure 4:**
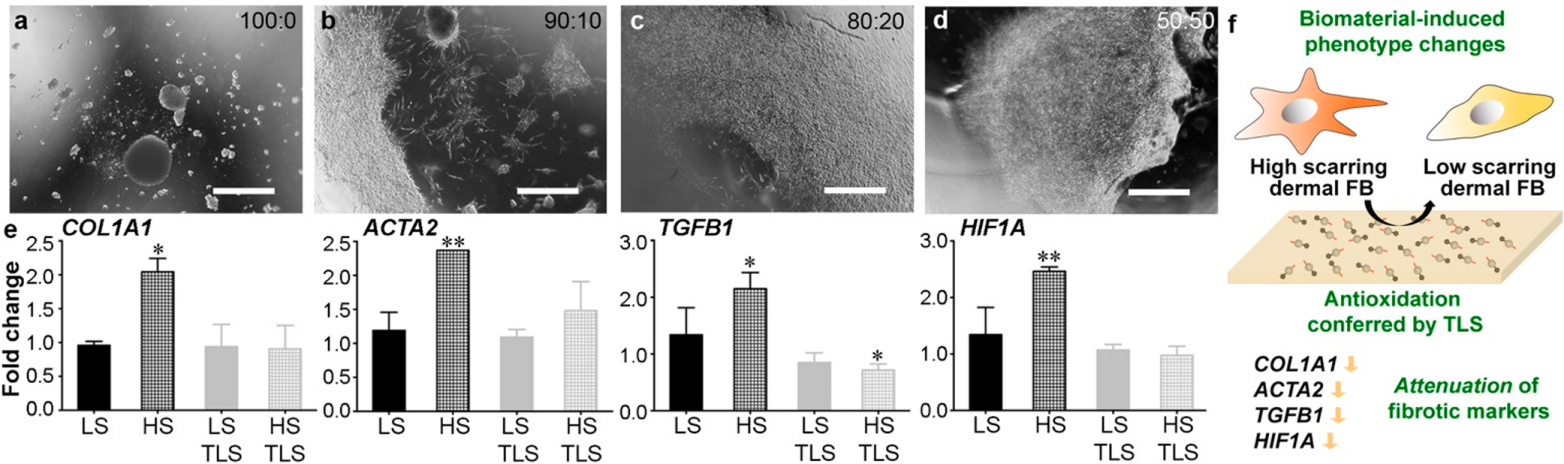
Attachment of hdFB to PEG composites and the modulation of fibrotic markers by TLS composites. Different ratios of PEG:fGelMA (v/v) composites (a) 100:0, (b) 90:10, (c) 80:20 and (d) 50:50, seeded with hdFBs for 48 h. Scale bar, 1000 μm. (e) LS (low scarring) and HS (high scarring) hdFBs were maintained in normal 2D cultures, while TLS denotes LS and HS hdFBs were cultured on top of PEG-fGelMA-TLS composites. Each hdFB phenotype contains two different donor hdFB cell lines. LS or HS, *n* = 2, mean ± SD; LS TLS or HS TLS, *n* = 3, mean ± SD. ANOVA Dunnett’s *post hoc* test, *p < 0.05 and **p < 0.01. (f) A scheme showing the potential mechanism of the phenotype changes conferred by TLS composites.

The goal of the current work is to test the antioxidant capacity of TLS in an engineered scaffold for tissue repair. Thus, we tested the alteration of fibrotic gene expression (*COL1A1*, *ACTA2*, *TGFB1* and *HIF1A*) by the PEG-fGelMA-TLS composites after 24 h. As shown in Figure 4e, all the fibrotic markers showed that the expression of each marker from HS hdFB was significantly higher than that of LS hdFB. When cultures of hdFBs were maintained on PEG-fGelMA-TLS scaffolds, the difference between two different hdFB cell lines was largely reduced. The resulting phenotype change induced by TLS composites is depicted in Figure 4f.

The expression of *COL1A1* on PEG-fGelMA-TLS composites was similar to LS FBs, which is the relatively proliferative phenotype. As a major component in collagen type I, *COL1A1* has been implicated in the overproduction of collagen and therefore increased fibrosis.^36^ In Figure 4e, the expression of *ACTA2* was significantly reduced on PEG-fGelMA-TLS composites and that of LS and HS FBs on PEG-fGelMA-TLS composites was similar (*p* = 0.923 with Tukey’s *post hoc* analysis). Elevated *ACTA* expression is attributed to differentiated myofibroblasts, which can be responsible for excess granulation tissues and fibrocontractive diseases. During the wound healing process, the expression of *ACTA1* and *ACTA2* is increased by contraction of the surrounding environment. Chronic or excessive contraction ultimately leads to deformation of the surrounding ECM network thus leading to further complications with the wound healing process or contracture, the permanent deformation of tissue.^37^ The expression of *TGFB1* in HS by PEG-fGelMA-TLS composites was significantly different from that of LS in normal tissue culture while the difference between LS and HS on PEG-fGelMA-TLS composites were not statistically significant. Overexpression of *TGFB1* ultimately leads to excess scar formation and reduced wound healing capacity.^37^ Additionally, adult wounds were found to contain higher expression of *TGFB1* when compared to fetal wounds, low scarring phenotype.^38−40^ The expression of *HIF1A* was diminished and comparable to that of LS. HIF1 regulates cell apoptosis and adapts to aid in cellular survival. The particular subunit, *HIF1A*, is regulated by oxygen and therefore under normal conditions is readily degraded.^41^ These results elucidate that PEG-fGelMA-TLS composites were able to remove the differential of hdFB responses associated with fibrosis over 24 h, which can be beneficial to enhancing the proliferation of HS hdFBs for regenerative medicine applications.

We sought to develop a precise and targeted method to formulate consistent composites with lignosulfonate. Thiol–ene click chemistry was utilized to synergistically form PEG-based TLS composites in an attempt to address the previous concerns over long-term stability. When using two uniquely different assays to observe the antioxidant capacity or scavenging potential, TLS exhibited more scavenging potentials than SLS due to the preservation of the phenolic component of SLS and thiolated aliphatic chains of SLS. Although these advantages are expected to attenuate fibrotic responses, long-term cultures can degrade fGelMA by enzymes and free TLS nanoparticles can be conjugated to cell membrane or internalized to cytoplasm. Concerns of cytotoxicity and immunogenicity from SLS were minimal as shown in our previous publication.^7^ However, disulfide formation between TLS and cell membrane or surrounding tissue warrants further investigations for tissue engineering applications. Because of the antiadhesion nature of PEG, we introduced fGelMA as a natural agent to enhance cell adhesion. The addition of fGelMA significantly increased cell adhesion of hdFBs with no phase separation of precursors. When cultured with hdFBs for 24 h, PEG-fGelMA-TLS composites were able to reduce the expression of key fibrotic genes in HS FBs to that of LS FBs. Consequently, the engineered PEG-fGelMA-TLS composites can be utilized as a cell culture platform exploiting sustainable materials of lignin and gelatin for enhanced wound healing applications.

## Supporting Information Available

Experimental procedures, photo-cross-linking scheme, ^1^H NMR and FT-IR spectra of SLS, TLS, and fGel, and comparison of viscosity of fGelMA to that of pGelMA (PDF)

## References

[ref1] Forman H. J.; Torres M. Reactive oxygen species and cell signaling: respiratory burst in macrophage signaling. Am. J. Respir. Crit. Care Med. 2002, 166 (12 Pt 2), S4–S8. 10.1164/rccm.2206007. 12471082

[ref2] Scherz-Shouval R.; Elazar Z. Regulation of autophagy by ROS: physiology and pathology. Trends Biochem. Sci. 2011, 36 (1), 30–8. 10.1016/j.tibs.2010.07.007. 20728362

[ref3] Finkel T.; Holbrook N. J. Oxidants, oxidative stress and the biology of ageing. Nature 2000, 408 (6809), 239–47. 10.1038/35041687. 11089981

[ref4] Sauer H.; Wartenberg M.; Hescheler J. Reactive oxygen species as intracellular messengers during cell growth and differentiation. Cell. Physiol. Biochem. 2001, 11 (4), 173–86. 10.1159/000047804. 11509825

[ref5] Dizhbite T.; Telysheva G.; Jurkjane V.; Viesturs U. Characterization of the radical scavenging activity of lignins--natural antioxidants. Bioresour. Technol. 2004, 95 (3), 309–17. 10.1016/j.biortech.2004.02.024. 15288274

[ref6] Baba S. A.; Malik S. A. Determination of total phenolic and flavonoid content, antimicrobial and antioxidant activity of a root extract of Arisaema jacquemontii Blume. Journal of Taibah University for Science 2015, 9 (4), 449–454. 10.1016/j.jtusci.2014.11.001.

[ref7] Belgodere J. A.; Zamin S. A.; Kalinoski R. M.; Astete C. E.; Penrod J. C.; Hamel K. M.; Lynn B. C.; Rudra J. S.; Shi J.; Jung J. P. Modulating Mechanical Properties of Collagen–Lignin Composites. ACS Applied Bio Materials 2019, 2 (8), 3562–3572. 10.1021/acsabm.9b00444. 35030742

[ref8] Quraishi S.; Martins M.; Barros A. A.; Gurikov P.; Raman S. P.; Smirnova I.; Duarte A. R. C.; Reis R. L. Novel non-cytotoxic alginate lignin hybrid aerogels as scaffolds for tissue engineering. J. Supercrit. Fluids 2015, 105, 1–8. 10.1016/j.supflu.2014.12.026.

[ref9] Yang W.; Fortunati E.; Bertoglio F.; Owczarek J. S.; Bruni G.; Kozanecki M.; Kenny J. M.; Torre L.; Visai L.; Puglia D. Polyvinyl alcohol/chitosan hydrogels with enhanced antioxidant and antibacterial properties induced by lignin nanoparticles. Carbohydr. Polym. 2018, 181, 275–284. 10.1016/j.carbpol.2017.10.084. 29253973

[ref10] Witzler M.; Alzagameem A.; Bergs M.; Khaldi-Hansen B. E.; Klein S. E.; Hielscher D.; Kamm B.; Kreyenschmidt J.; Tobiasch E.; Schulze M. Lignin-Derived Biomaterials for Drug Release and Tissue Engineering. Molecules 2018, 23 (8), 1885 10.3390/molecules23081885. PMC6222784 30060536

[ref11] Kai D.; Zhang K. Y.; Jiang L.; Wong H. Z.; Li Z. B.; Zhang Z.; Loh X. J. Sustainable and Antioxidant Lignin-Polyester Copolymers and Nanofibers for Potential Healthcare Applications. ACS Sustainable Chem. Eng. 2017, 5 (7), 6016–6025. 10.1021/acssuschemeng.7b00850.

[ref12] Kai D.; Ren W.; Tian L. L.; Chee P. L.; Liu Y.; Ramakrishna S.; Loh X. J. Engineering Poly(lactide)-Lignin Nanofibers with Antioxidant Activity for Biomedical Application. ACS Sustainable Chem. Eng. 2016, 4 (10), 5268–5276. 10.1021/acssuschemeng.6b00478.

[ref13] Sghayyar H. N. M.; Lim S. S.; Ahmed I.; Lai J. Y.; Cheong X. Y.; Chong Z. W.; Lim A. F. X.; Loh H. S. Fish biowaste gelatin coated phosphate-glass fibres for wound-healing application. Eur. Polym. J. 2020, 122, 109386 10.1016/j.eurpolymj.2019.109386.

[ref14] Zhou L. P.; Xu T. W.; Yan J. C.; Li X.; Xie Y. Q.; Chen H. Fabrication and characterization of matrine-loaded konjac glucomannan/fish gelatin composite hydrogel as antimicrobial wound dressing. Food Hydrocolloids 2020, 104, 105702 10.1016/j.foodhyd.2020.105702.

[ref15] Zhang X.; Kim G. J.; Kang M. G.; Lee J. K.; Seo J. W.; Do J. T.; Hong K.; Cha J. M.; Shin S. R.; Bae H. Marine Biomaterial-Based Bioinks for Generating 3D Printed Tissue Constructs. Mar. Drugs 2018, 16 (12), 484 10.3390/md16120484. PMC6315353 30518062

[ref16] Yoon H. J.; Shin S. R.; Cha J. M.; Lee S. H.; Kim J. H.; Do J. T.; Song H.; Bae H. Cold Water Fish Gelatin Methacryloyl Hydrogel for Tissue Engineering Application. PLoS One 2016, 11 (10), e0163902 10.1371/journal.pone.0163902. 27723807 PMC5056724

[ref17] Gómez-Guillén M. C.; Pérez-Mateos M.; Gómez-Estaca J.; López-Caballero E.; Giménez B.; Montero P. Fish gelatin: a renewable material for developing active biodegradable films. Trends Food Sci. Technol. 2009, 20 (1), 3–16. 10.1016/j.tifs.2008.10.002.

[ref18] Chiou B. S.; Avena-Bustillos R. J.; Bechtel P. J.; Jafri H.; Narayan R.; Imam S. H.; Glenn G. M.; Orts W. J. Cold water fish gelatin films: Effects of cross-linking on thermal, mechanical, barrier, and biodegradation properties. Eur. Polym. J. 2008, 44 (11), 3748–3753. 10.1016/j.eurpolymj.2008.08.011.

[ref19] Chiou B. S.; Avena-Bustillos R. J.; Shey J.; Yee E.; Bechtel P. J.; Imam S. H.; Glenn G. M.; Orts W. J. Rheological and mechanical properties of cross-linked fish gelatins. Polymer 2006, 47 (18), 6379–6386. 10.1016/j.polymer.2006.07.004.

[ref20] Vandooren J.; Van den Steen P. E.; Opdenakker G. Biochemistry and molecular biology of gelatinase B or matrix metalloproteinase-9 (MMP-9): the next decade. Crit. Rev. Biochem. Mol. Biol. 2013, 48 (3), 222–72. 10.3109/10409238.2013.770819. 23547785

[ref21] Liu H. L.; Chung H. Y. Self-Healing Properties of Lignin-Containing Nanocomposite: Synthesis of Lignin-graft-poly(5-acetylaminopentyl acrylate) via RAFT and Click Chemistry. Macromolecules 2016, 49 (19), 7246–7256. 10.1021/acs.macromol.6b01028.

[ref22] Han Y. M.; Yuan L.; Li G. Y.; Huang L. H.; Qin T. F.; Chu F. X.; Tang C. B. Renewable polymers from lignin via copper-free thermal click chemistry. Polymer 2016, 83, 92–100. 10.1016/j.polymer.2015.12.010.

[ref23] Kolb H. C.; Finn M. G.; Sharpless K. B. Click Chemistry: Diverse Chemical Function from a Few Good Reactions. Angew. Chem., Int. Ed. 2001, 40 (11), 2004–2021. 10.1002/1521-3773(20010601)40:11<2004::AID-ANIE2004>3.3.CO;2-X. 11433435

[ref24] Hoyle C. E.; Bowman C. N. Thiol-ene click chemistry. Angew. Chem., Int. Ed. 2010, 49 (9), 1540–73. 10.1002/anie.200903924. 20166107

[ref25] Lowe A. B. Thiol-ene ″click″ reactions and recent applications in polymer and materials synthesis: a first update. Polym. Chem. 2014, 5 (17), 4820–4870. 10.1039/C4PY00339J.

[ref26] Liu H. L.; Chung H. Y. Visible-Light Induced Thiol-Ene Reaction on Natural Lignin. ACS Sustainable Chem. Eng. 2017, 5 (10), 9160–9168. 10.1021/acssuschemeng.7b02065.

[ref27] Jacob C. A scent of therapy: pharmacological implications of natural products containing redox-active sulfur atoms. Nat. Prod. Rep. 2006, 23 (6), 851–63. 10.1039/b609523m. 17119635

[ref28] An S.; Jeon B.; Bae J. H.; Kim I. S.; Paeng K.; Kim M.; Lee H. Thiol-based chemistry as versatile routes for the effective functionalization of cellulose nanofibers. Carbohydr. Polym. 2019, 226, 115259 10.1016/j.carbpol.2019.115259. 31582070

[ref29] Liu L. Y.; Hua Q.; Renneckar S. A simple route to synthesize esterified lignin derivatives. Green Chem. 2019, 21 (13), 3682–3692. 10.1039/C9GC00844F.

[ref30] Hoch E.; Schuh C.; Hirth T.; Tovar G. E.; Borchers K. Stiff gelatin hydrogels can be photo-chemically synthesized from low viscous gelatin solutions using molecularly functionalized gelatin with a high degree of methacrylation. J. Mater. Sci.: Mater. Med. 2012, 23 (11), 2607–17. 10.1007/s10856-012-4731-2. 22890515

[ref31] Nguyen A. H.; McKinney J.; Miller T.; Bongiorno T.; McDevitt T. C. Gelatin methacrylate microspheres for controlled growth factor release. Acta Biomater. 2015, 13, 101–10. 10.1016/j.actbio.2014.11.028. 25463489 PMC4293288

[ref32] Liang J.; Guo Z.; Timmerman A.; Grijpma D.; Poot A. Enhanced mechanical and cell adhesive properties of photo-crosslinked PEG hydrogels by incorporation of gelatin in the networks. Biomed Mater. 2019, 14 (2), 024102 10.1088/1748-605X/aaf31b. 30524039

[ref33] Hutson C. B.; Nichol J. W.; Aubin H.; Bae H.; Yamanlar S.; Al-Haque S.; Koshy S. T.; Khademhosseini A. Synthesis and characterization of tunable poly(ethylene glycol): gelatin methacrylate composite hydrogels. Tissue Eng., Part A 2011, 17 (13–14), 1713–23. 10.1089/ten.tea.2010.0666. 21306293 PMC3118706

[ref34] Dean J. C.; Navotnaya P.; Parobek A. P.; Clayton R. M.; Zwier T. S. Ultraviolet spectroscopy of fundamental lignin subunits: guaiacol, 4-methylguaiacol, syringol, and 4-methylsyringol. J. Chem. Phys. 2013, 139 (14), 144313 10.1063/1.4824019. 24116625

[ref35] Deptuła P.; Łysik D.; Pogoda K.; Cieśluk M.; Namiot A.; Mystkowska J.; Król G.; Głuszek S.; Janmey P. A.; Bucki R. Tissue Rheology as a Possible Complementary Procedure to Advance Histological Diagnosis of Colon Cancer. ACS Biomater. Sci. Eng. 2020, 6 (10), 5620–5631. 10.1021/acsbiomaterials.0c00975. 33062848 PMC7549092

[ref36] Ma H. P.; Chang H. L.; Bamodu O. A.; Yadav V. K.; Huang T. Y.; Wu A. T. H.; Yeh C. T.; Tsai S. H.; Lee W. H. Collagen 1A1 (COL1A1) Is a Reliable Biomarker and Putative Therapeutic Target for Hepatocellular Carcinogenesis and Metastasis. Cancers 2019, 11 (6), 786 10.3390/cancers11060786. PMC6627889 31181620

[ref37] Tomasek J. J.; Gabbiani G.; Hinz B.; Chaponnier C.; Brown R. A. Myofibroblasts and mechano-regulation of connective tissue remodelling. Nat. Rev. Mol. Cell Biol. 2002, 3 (5), 349–63. 10.1038/nrm809. 11988769

[ref38] Burrington J. D. Wound healing in the fetal lamb. J. Pediatr Surg 1971, 6 (5), 523–8. 10.1016/0022-3468(71)90373-3. 5166565

[ref39] Rowlatt U. Intrauterine wound healing in a 20 week human fetus. Virchows Arch. A: Pathol. Anat. Histol. 1979, 381 (3), 353–61. 10.1007/BF00432477. 155931

[ref40] Sen C. K.; Gordillo G. M.; Roy S.; Kirsner R.; Lambert L.; Hunt T. K.; Gottrup F.; Gurtner G. C.; Longaker M. T. Human skin wounds: a major and snowballing threat to public health and the economy. Wound Repair Regen 2009, 17 (6), 763–71. 10.1111/j.1524-475X.2009.00543.x. 19903300 PMC2810192

[ref41] Distler J. H.; Jüngel A.; Pileckyte M.; Zwerina J.; Michel B. A.; Gay R. E.; Kowal-Bielecka O.; Matucci-Cerinic M.; Schett G.; Marti H. H.; Gay S.; Distler O. Hypoxia-induced increase in the production of extracellular matrix proteins in systemic sclerosis. Arthritis Rheum. 2007, 56 (12), 4203–15. 10.1002/art.23074. 18050252

