# Supplementary material for: Attenuating Fibrotic Markers of Patient-Derived Dermal Fibroblasts by Thiolated Lignin Composites
Source: ACS Biomater Sci Eng. 2021 May 3;7(6):2212–8. doi: 10.1021/acsbiomaterials.1c00427 (PMC8290399; doi:10.1021/acsbiomaterials.1c00427)
Supplement: Supplementary file 1 — ab1c00427_si_001.pdf [file ab1c00427_si_001.pdf]

Supporting Information

Attenuating fibrotic markers of patient-derived dermal fibroblasts by thiolated lignin composites

Jorge A. Belgodere[1,$], Dongwan Son[2,$], Bokyoung Jeon[1,2,], Jongwon Choe[1,2], Anna C. Guidry[1], Adam X. Bao[1], Syed A. Zamin[1], Umang M. Parikh[3], Swathi Balaji[3], Myungwoong Kim[2*], Jangwook P. Jung[1*,]

[1]Department of Biological Engineering, Louisiana State University, Baton Rouge, LA
[2]Department of Chemistry and Chemical Engineering, Inha University, Incheon 22212, Republic of Korea
[3]Department of Pediatric Surgery, Texas Children's Hospital and Baylor College of Medicine, Houston, TX

[$]co-first author

KEYWORDS: antioxidation, fish gelatin, lignosulfonate, fibrosis, wound healing

Number of pages: 10
Number of figures: 5
Number of tables: 0

MATERIALS AND METHODS

Thiolation of sodium lignosulfonate

To produce thiolated lignosulfonate (TLS), sodium lignosulfonate (SLS, TCI Chemicals, Cat#L0098, Lot#V5VJF-IC, >94% purity) was functionalized with thiol groups by acid-catalyzed esterification (Figure 1). For thiolation, 1 g of SLS was dissolved in 10 mL of Milli-Q water in an Erlenmeyer flask, then 1 mL of 3-mercaptopropionic acid (TCI Chemicals, Cat#M0061) and 0.1 mL of hydrochloric acid (Fisher scientific, 37% purity, Cat#A144S-500) were added to the solution. The flask was purged with nitrogen gas and placed in an oil bath at 80°C. After 24 h, the flask was removed and filled with 100 mL of isopropyl alcohol (IPA, VWR, Cat#700002-608) until the TLS precipitated. The mixture was then transferred to 50 mL conical tubes, centrifuged at 4300 RCF for 10 min. The supernatant removed was removed and replaced with fresh IPA, which was repeated two more times. After this washing step was completed, solid TLS was separated using a Buchner funnel and vacuum dried for 24 h.

Ellman's assay for quantification of thiolation

The degree of thiolation was determined using Ellman's assay (5,5'-dithiobis(2-nitrobenzoic acid) (Ellman's reagent, Acros Organics, 99% purity, Cat#AC117540010), following the manufacturer's protocol. Briefly, the reaction buffer was made by dissolving 2.130 g of sodium phosphate (Acros Organics, Cat# AC204851000) and 43.5 mg of ethylenediaminetetraacetic acid (EDTA, TCI Chemicals, Cat# E0084) in 150 mL of phosphate buffer saline (PBS, Fisher Scientific, Cat# BP399-4). In an Eppendorf tube, Ellman's reagent was dissolved in the reaction buffer to create a 4 mg/mL solution. In separate Eppendorf tubes, TLS and SLS were also dissolved in the reaction buffer. Using *L*-cysteine hydrochloride monohydrate (Alfa Aesar, 99% purity, Cat#A10389-14) a serial dilution was prepared to create a standard curve. In separate Eppendorf tubes, 125 µL of either the standards or unknowns, 25 µL of the Ellman's reagent, and 1.25 mL of the reaction buffer were mixed. The solutions were then pipetted into a 96-well plate and

incubated at room temperature for 30 min. Absorbance was monitored at 412 nm and thiol concentrations were calculated using the standard curve.

<u>Methacrylation of fish gelatin (fGelMA) and porcine gelatin (pGelMA)</u>

fGelMA was prepared by the reaction of primary amine in gelatin with glycidyl methacrylate (GMA, Sigma, Cat# 151238)[1]. In 100 mL of DMSO (DMSO, TCI Chemicals, Cat# D0798), 10 g of gelatin from cold water skin (fGel, Sigma, Cat# G7041) was added and stirred at 50°C for 30 min until the fGel is fully dissolved. Then, 4 mL of GMA and 0.6 g of 4-(dimethylamino)pyridine (DMAP, Alfa Aesar, Cat# A13016) were slowly added to the fGel solution, followed by further stirring at 50°C for 2 days. The reaction mixture was dialyzed against DI water at 40°C using dialysis tubes (Spectra/Por 3, Spectrum™, 3500 Da molecular weight cut-off (MWCO)) for 5 days. The dialyzed fGelMA solution was lyophilized over 4 days.

For synthesizing pGelMA, in a scintillation vial, 1.0 g of porcine skin gelatin (Type A, MP Biomedicals, Cat#901771) is dissolved in 10 mL Phosphate Buffer Saline (PBS) at 50°C. Additional 0.1782 g of 1-Ethyl-3-(3-dimethylaminopropyl) carbodiimide (EDC, TCI, Cat#D1601), 0.1070 g of N-Hydroxysuccinimide (NHS, TCI, Cat#H0623) were dissolved in 2.0 mL of dimethyl sulfoxide (DMSO, MP Biomedicals, Cat#196055). Next, 0.1 mL of methacrylic acid (MA, Acros Organics, Cat#1683-2500) was added, dropwise, to the DMSO solution, a stir bar was added, and placed into an oil bath at 40°C for 30 min. After 30 min, MA solution was added, dropwise, to the gelatin solution with a stir bar. The GelMA solution was placed into the oil bath at 50°C for 1.5 h. This reaction introduces methacryloyl substitution on the amine of the amino acid residues of gelatin. Once the reaction was completed, GelMA was placed into dialysis tubing (Spectra/Por 1 Dialysis Membrane, 6-8 kDa, Cat#132655T), then placed into a beaker with Milli-Q water (>18.2 Ω·cm), and then onto a hot plate at 40°C for 1 week. Water was exchanged twice a day for the entire duration. Dialysis allowed for the complete removal of the low-molecular-weight impurities (including unreacted MA and MA byproducts and DMSO), which are potentially cytotoxic. Finally,

the dialyzed solution was lyophilized (Freezone Model 77530, Labconco) for 3 days and stored at 4°C until use. The degree of substitution was calculated using ^{1}H nuclear magnetic resonance (NMR), yielding about 87%.

Spectroscopic characterizations

The degree of functionalization of TLS was determined using quantitative nuclear magnetic resonance (NMR) spectroscopy with ^{1}H and ^{31}P NMR (JNMECZ400S 400 MHz, JEOL), following the literature[2, 3]. Cyclohexanol was added as an internal standard for quantification (145.0 ppm) for ^{31}P NMR. Comparing integrated peak area of the standard to areas of aliphatic OH and phenolic OH, the degree of the functionalization was determined. ^{1}H NMR spectrum of fGelMA was acquired in deuterium oxide (D_2O, Cambridge Isotope Laboratories, Inc., DLM-4-25). Fourier-transform infrared (FT-IR) spectra of SLS and TLS were recorded on a Perkin Elmer Frontier equipped with a UATR unit in the wavenumber range of from 4000 to 640 cm^{-1} at room temperature.

Formation of PEG-fGelMA composite

PEG composites were formed by weighing out PEG-diacrylate (PEGDA, Laysan bio, Cat# ACRL-PEG-ACRL-10K-5g), lithium phenyl-2,4,6-trimethylbenzoylphosphinate (LAP, Cat# LAP, Allevi), and TLS. The final concentrations of PEG and LAP were 40 and 5 mg/mL, respectively. TLS concentration was varied based on molar ratios between the acrylate groups on the PEGDA and thiol groups on the lignin, fixed at 1:0.5 (ene:thiol). To enhance cellular adhesion, 200 mg/mL fGelMA and 5 mg/mL LAP solutions were added to the PEG solution. The optimal ratio of PEG to fGelMA was 80:20 (v/v). Solutions were pipetted into custom molds and UV crosslinked using a UV floodlamp (Intelli-Ray 400, Uvitron international) for 120 s at 10 mW/cm^2.

Oscillating rheometry

Using a TA Discovery HR-2 rheometer, viscosity was measured in a flow ramp setting (shear rate from 1 to 100 (1/s)) and with a 25 mm parallel plate. Using an 8 mm parallel plate, storage (G′) and loss (G″) moduli of composites were determined by frequency sweeping from 0.62 to 19.9 (rad/s) at 2% strain. In addition, instead of evaluating stiffness at an arbitrary storage modulus (often storage moduli are altered by axial stress applied during measurement), we evaluated the slope of axial stress vs compression, similar to evaluating Young's modulus from the slope of a stress-strain curve[4]. Axial stresses at 0, 10 and 20% of compression were determined, while composite samples were subject to 2% strain and 6.28 rad/s frequency.

<u>DPPH assay</u>

The antioxidant activity of SLS and TLS was evaluated using the 2,2-dipehnyl-1-picrylhydrazyl (DPPH, Alfa Aesar, Cat# 44150) radical scavenging assay. Briefly, a 0.2 mM DPPH solution was prepared in 1:1 mixture of ethanol (Fischer, Cat# BP2818-500, 200 proof) and water (Fisher, Cat# W2-4) since SLS or TLS is not completely soluble in ethanol. DPPH solution without sample was used as the control. After incubating samples in darkness at room temperature for 30 min and 24 h with mild agitation of 150 rpm, the decreases in solution absorbance was measured at 517 nm using a Cytation3 (Biotek) spectrophotometer. *L*-ascorbic acid (Sigma, Cat# A4403-100MG) was used as a positive control. Absorbance of all samples without DPPH was subtracted to correct the background absorbance at 517 nm. The DPPH radical scavenging activity (%) was calculated using the following formula: DPPH radical scavenging activity (%) = $(A_c – A_s)/A_c \times 100$ (%), where A_c is absorbance of control and A_s is absorbance of samples.

<u>TAC assay (antioxidant activity assay)</u>

The total antioxidant capacity (TAC) of SLS and TLS was confirmed by evaluating the free radical scavenging effect using the Oxiselect™ TAC assay kit (Cat# STA-360, Cell Biolabs). Briefly, all reagents and a uric acid standard were prepared following the provided protocol.

Samples were prepared with the reaction buffer and the copper ion solution and allowed to react at room temperature for 5 min. After 5 min, the quenching reagent was added and 100 µL was then transferred to a 96-well plate. The uric acid standard curve was prepared following the provided protocol and 100 µL was pipetted into the 96-well plate. Absorbance at 490 nm was measured using a Cytation 3 (Biotek) spectrophotometer. Absorbance of all samples in the reaction buffer was subtracted to correct the background absorbance at 490 nm. Using the linear regression from standard curve samples, uric acid equivalent (UAE)/mL for each sample was calculated.

<u>Cultures of patient-derived dermal fibroblasts</u>

Skin tissue was collected from abdominoplasty patients who provided written informed consent as part of a protocol approved by the Institutional Review Board of Texas at Baylor College of Medicine (Approval # H-38187) in accordance with the Declaration of Helsinki. A biobank of scar and matched normal uninjured skin tissue was obtained from abdominoplasty patients controlled for sex, age, ethnicity, surgery type, indication, wound site, and comorbidities. These skin tissues were grouped into low scarring (LS) and high scarring (HS) phenotypes based on the evaluation of their existing C-section scars using Vancouver Scar Scale (VSS). Skin obtained from patients with 1-3 score on VSS were categorized as LS phenotype (N). For each group, n=3 skin tissues were pooled. Only LS- and HS-normal (N) fibroblasts were used for cell studies and two donor cell lines were used for each hdFB phenotypes. Both LS-N and HS-N human dermal fibroblasts (dFBs) were maintained in DMEM (Cat# 10567-014-500mL, Gibco) supplemented with 10% fetal bovine serum (FBS, Cat# 35-015-CV, Corning), Penicillin/Streptomycin (P/S, Cat# 15140-122, Gibco), and antibiotic/antimycotic (anti/anti, Cat#: 15240-062, Gibco). Only passages 8-10 were used for studies and cells were harvested at around 80% confluency with TrypLE Express (Cat#: 12604-021, Gibco) application and pelleted at 350 g

for 5 min. Supernatant was removed and hdFBs were gently resuspended in cell culture media and counted. Media was changed every other day.

Quantitative Reverse Transcriptase Polymerase Chain Reaction (qRT-PCR)

LS-N or HS-N hdFBs were seeded onto PEG-fGelMA-TLS composites at 50,000 cells/cm^2. Cell culture supernatants were collected from all of the gels and control wells at 24 h after culture initiation and stored in -80°C with protease inhibitor cocktail (Sigma). At specified time points cell-seeded composites were removed and incubated with the application of TrypLE Express (Cat#: 12604-021, Gibco) to generate a cell suspension. RNA was extracted following the suggested procedure from the PureLink RNA MicroPrep kit (Cat#: 12183018A, ThermoFisher Scientific). Extracted RNA was evaluated for quantity and purity using a Take3 Micro-Volume plate (Biotek) and Cytation3 spectrophotometer (Biotek). cDNA was reverse-transcribed using a High-Capacity RNA-to-cDNA kit (Cat#: 4387406, Applied Biosystems) following manufacturer's protocols. Primers for COL1A1, TGFB1 and GAPDH were purchased from Bio-Rad and those for ACTA2 (ASMA, FOR: 5′ ACCCACAATGTCCCCATCTA 3′, REV: 5′ GAAGGAATAGCCACGCTCAG 3′) and HIF1A (FOR: 5′ GATGTAATGCTCCCCTCACC 3′, REV: 5′ CTTGATTGAGTGCAGGGTCA 3′) were purchased from Millipore-Sigma. qRT-PCR was performed using Power SYBR Green PCR Master Mix (Cat# 4367659, Applied Biosystem) in Bio-Rad CFX 384 Real-Time system. Relative mRNA levels were calculated using the $2^{-\Delta\Delta CT}$ method and normalized to GAPDH. The gene expression in 2D cultures was normalized to one of the two cell line and that of 3D culture was normalized to PEG-fGelMA controls (without TLS).

Supporting Figures

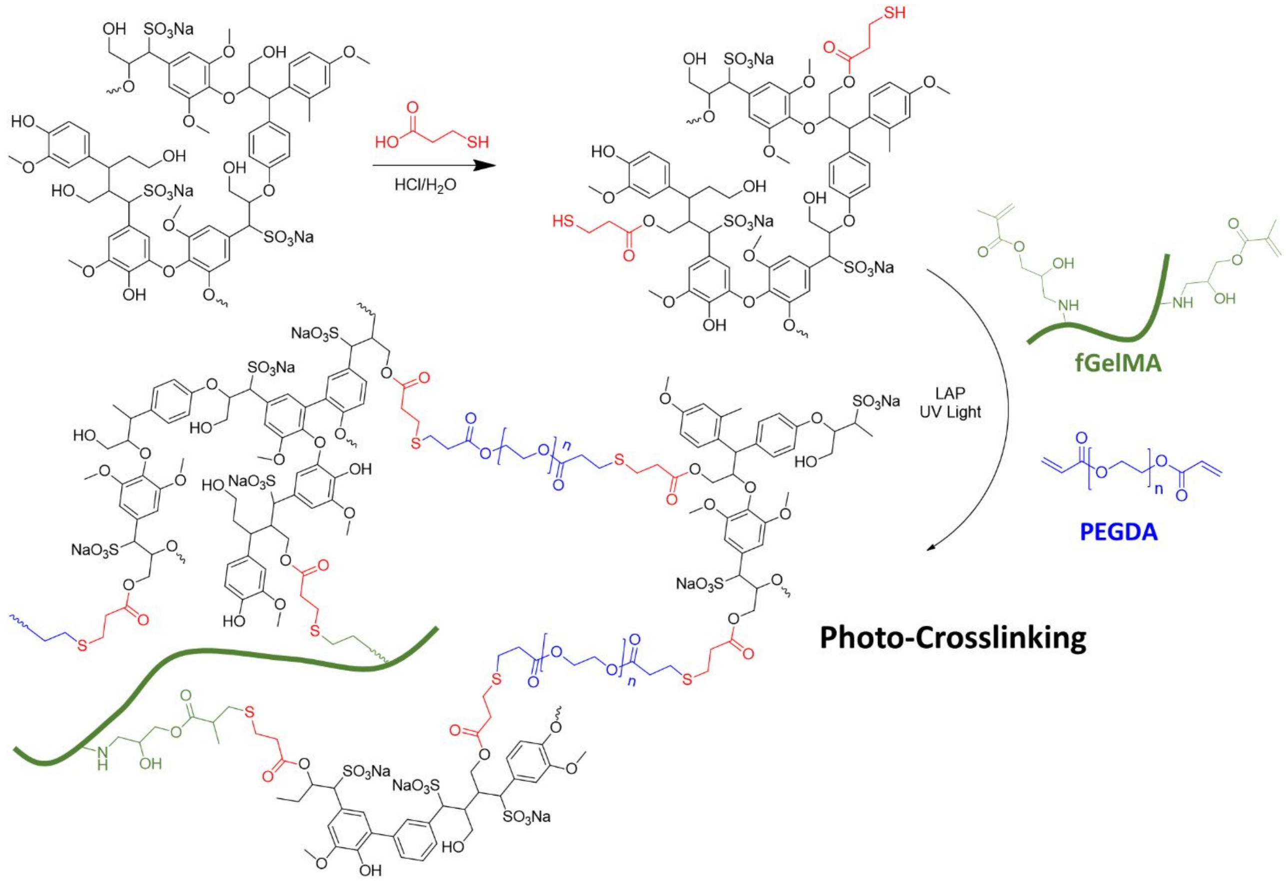

Figure S1. Scheme showing the chemical route of thiol-ene photo-crosslinking of TLS, PEGDA and fGelMA via radical process. Note that the esterification sites and their number were arbitrarily assigned due to the structural complexity of lignosulfonate.

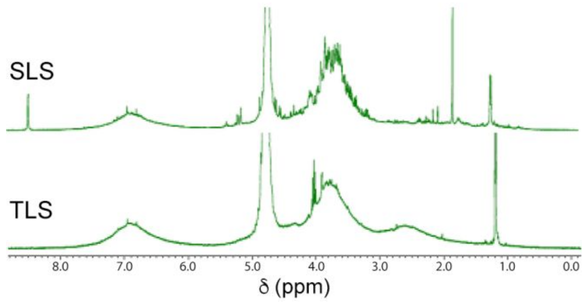

Figure S2. **^{1}H NMR spectra of SLS and TLS.** ^{1}H NMR spectra were normalized with the peak of δ 5.6-8.2 ppm, which is for aromatic proton. In comparison with the SLS, ^{1}H NMR spectrum of TLS roughly confirmed the functionalization by the appearance of a broad peaks that corresponded to protons of CH_2 groups adjacent to thiol (δ 1.9-3.2 ppm). The chemical shifts for protons in aliphatic groups (δ 2.8-4.3 ppm) reduced after the functionalization.

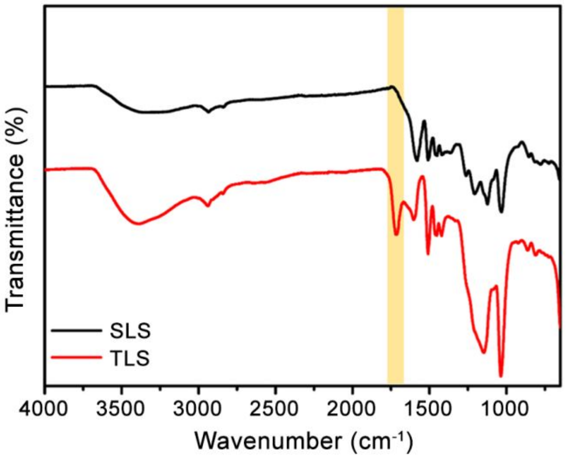

Figure S3. **FT-IR spectra of SLS and TLS.** The emergence of the intense band at about 1700 cm^{-1} assigned to stretch mode of the C=O in ester, which is formed upon the reaction of carboxylic acid in MPA to alcohol in SLS, confirm the successful esterification reaction.

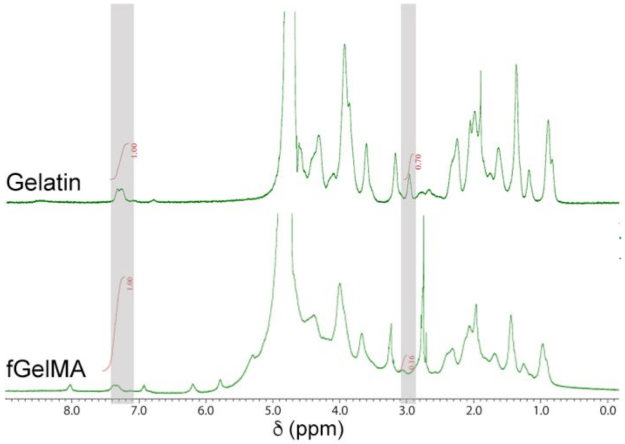

Figure S4. Assessment of the methacrylation of fGel. ^{1}H NMR spectra of pristine fGel and fGelMA in D_2O. The incorporation of methacrylate was confirmed by the decrease in the signal from the primary amine of lysine and the emergence of the signal from alkene in the methacrylate.

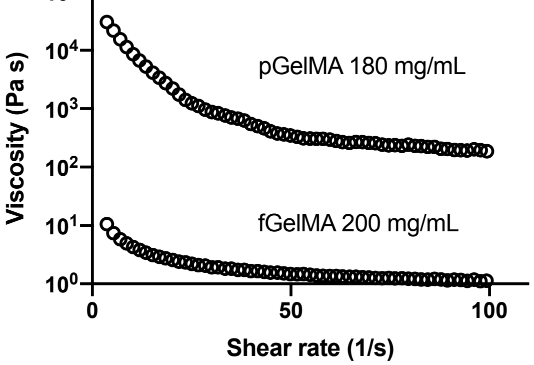

Figure S5. Comparison of viscosity of fGelMA to that of pGelMA. The viscosity of pGelMA at 180 mg/mL and fGelMA at 200 mg/mL was measured by oscillating rheometry. pGelMA at 180 mg/mL is the highest concentration that can be prepared in liquid.

REFERENCES

1. Wang, Z.; Tian, Z.; Menard, F.; Kim, K., Comparative study of gelatin methacrylate hydrogels from different sources for biofabrication applications. *Biofabrication* **2017,** *9* (4), 044101.
2. Liu, L. Y.; Hua, Q.; Renneckar, S., A simple route to synthesize esterified lignin derivatives. *Green Chem.* **2019,** *21* (13), 3682-3692.
3. Jin, C.; Zhang, X.; Xin, J.; Liu, G.; Wu, G.; Kong, Z.; Zhang, J., Clickable synthesis of 1,2,4-triazole modified lignin-based adsorbent for the selective removal of Cd(II). *ACS Sustain. Chem. Eng.* **2017,** *5* (5), 4086-4093.
4. Deptuła, P.; Łysik, D.; Pogoda, K.; Cieśluk, M.; Namiot, A.; Mystkowska, J.; Król, G.; Głuszek, S.; Janmey, P. A.; Bucki, R., Tissue Rheology as a Possible Complementary Procedure to Advance Histological Diagnosis of Colon Cancer. *ACS Biomater Sci Eng* **2020,** *6* (10), 5620-5631.
